# Emergence of highly infectious SARS-CoV-2 variants in Bangladesh: the need for systematic genetic surveillance as a public health strategy

**DOI:** 10.1186/s41182-021-00360-w

**Published:** 2021-09-01

**Authors:** Mohammad Mehedi Hasan, Ian Christopher N. Rocha, Kimberly G. Ramos, Trisha Denise D. Cedeño, Ana Carla dos Santos Costa, Christos Tsagkaris, Md. Masum Billah, Shoaib Ahmad, Mohammad Yasir Essar

**Affiliations:** 1 Department of Biochemistry and Molecular Biology, Faculty of Life Science, Mawlana Bhashani Science and Technology University, Tangail, Bangladesh; 2 Division of Infectious Diseases, The Red-Green Research Centre, BICCB, Dhaka, Bangladesh; 3 School of Medicine, Centro Escolar University, Manila, Philippines; 4 Faculty of Medicine, Federal University of Bahia, Salvador, Bahia Brazil; 5 Faculty of Medicine, University of Crete, Heraklion, Greece; 6 Department of Pharmacy, Faculty of Life Science, Mawlana Bhashani Science and Technology University, Tangail, Bangladesh; 7 Punjab Medical College, Faisalabad, Pakistan; 8 Medical Research Center, Kateb University, 1001 Kabul, Afghanistan

**Keywords:** Bangladesh, SARS-CoV-2 variants, Public health, Genetic surveillance

## Abstract

Bangladesh, a low-middle-income country in South Asia is facing one of its worst public health emergencies due to the COVID-19 pandemic. The increase in the number of cases from the disease, since the second half of March 2021, can potentially cause the health system overload, and has, as one of the main reasons, the non-compliance with measures of social distance and the emergence of the variants of concern in the country. This increase in the contagion curve can also provide a favorable environment for the occurrence of more mutations in the structure and genome of the virus. Therefore, there is an urge to carry out genomic surveillance programs in order to identify, monitor and characterize these variants, and understand whether the vaccines currently used are effective against them.

Dear editor,

The ongoing Coronavirus Disease 2019 (COVID-19) pandemic is a major burden to health systems worldwide, constituting the worst health crisis in history. For Bangladesh, a low-middle-income country in South Asia, the situation was no different. As of July 20, 2021, 1,117,310 cases of COVID-19 had already been diagnosed in the country, with 154,087 active cases, 11,578 daily new cases, and 18,125 deaths [1]. With only 0.94% of its population having received at least the first dose of the COVID-19 vaccine as of July 18, 2021, a percentage that is lower than other countries, Bangladesh has experienced a drastic increase in the number of cases and fatalities [2].

In fact, according to Bangladesh’s Directorate General of Health Services, the number of COVID-19 cases in the country began to increase during the middle of March 2021. Initially, there was a drop in the number of cases during February 2021, in which the lowest monthly infection rate of 2.82% was observed. However, the numbers spiked up to 10.39% within the following month [3]. Moreover, the surge in COVID-19 infections can be attributed to the public’s ease and lack of compliance to safety measures and health guidelines. Experts also pointed out that the recent emergence in the country of highly infectious strains of the virus may also be responsible for this recent dramatic increase in the number of cases [4].

In this context, Bangladeshi health experts emphasized the need to detect the novel variants of the severe acute respiratory syndrome coronavirus 2 (SARS-CoV-2) that entered the country. As of this writing, the variants found in Bangladesh are the B.1.1.7 (Alpha) variant from the United Kingdom (UK), the B.1.351 (Beta) variant from South Africa, the P.1 (Gamma) variant from Brazil, and the B.1.617 (Delta) variant from India which are all considered as variants of concern (VOC) by the World Health Organization and the Centers for Disease Control and Prevention (CDC) [5]. During April to May 2021, the Beta variant accounted for 90% of all variants prevailing in Dhaka [5]. However, when the Delta variant arrived in Bangladesh in early May 2021, it had become the most prominent variant constituting 68% of the variants circulating in the Dhaka city by the end of May 2021 [5].

As stated by the CDC, these VOCs are proven to be highly contagious, transmissible, and alarmingly more severe due to increasing rates of hospitalizations and mortality [6]. In addition, VOCs have substantial evidence of reduced neutralization of antibodies produced during previous infection or inoculation, low responsiveness to therapeutic treatment and medication, less efficacy to vaccines, and failure in diagnostic detection [6]. Due to these attributes, the emergence of VOCs in Bangladesh may be one of the factors responsible for the exponential increase in the number of COVID-19 cases in the country in a short period of time, despite the implementation of preventive and control measures by the national government and vaccination [7].

To determine if the current trend of increasing COVID-19 cases is due to the new foreign or locally evolved variants, it is necessary to keep track of the mutations by genetic surveillance. Before the COVID-19 pandemic, genetic surveillance was reserved mainly for conducting small studies in antibiotic-resistant bacteria, investigating outbreaks, and monitoring influenza strains [8]. Unfortunately, this new testing strategy is not yet being implemented to detect and monitor novel variants of SARS-CoV-2 in the country. Since March 2020, most laboratories in Bangladesh only use the generic and typical reverse transcription-polymerase chain reaction (RT-PCR) test for COVID-19. However, the standard RT-PCR test is insufficient to definitively identify SARS-CoV-2 variants, as viruses are constantly evolving, and the genetic variations that occur over time can lead to the emergence of new variants, which may have different characteristics [8].

Therefore, it is essential to monitor circulating viruses for key mutations that happen in important regions of the genome. With the recent emergence of novel variants, laboratories in some countries started to use genetic surveillance, which involves genetic sequencing that decodes the genome of SARS-CoV-2 in samples from patients, allowing consistent and coordinated screening of positive samples [9]. Although more costly and time-consuming than other tests, full sequencing of viral genomes is a cornerstone of surveillance efforts because it helps scientists to identify and characterize variants present in a community according to transmissibility and disease severity. This is essential in developing diagnostic protocols, therapeutics, and vaccines, as well as formulating action plans to mitigate transmission [8, 9]. Moreover, genomic surveillance is necessary, as the rapid surge of COVID-19 cases in Bangladesh may provide a favorable environment for the occurrence of more mutations in the viral structure and genome, resulting in the emergence of a more infectious local variant.

Hence, at the present time, genomic surveillance is one of the main weapons for the early detection of new variants of SARS-CoV-2 in Bangladesh. The fact is that, detecting variants of concern and developing an evidence-based and timely public health response to mitigate the spread of the new variants, requires a robust genomic surveillance program. That translates to scientists sequencing virus samples from about 5% of the total number of COVID-19 patients, selected to be representative of the populations most at risk from the disease [8]. Without genomic surveillance, new variants may spread uncontrollably and may be undetected in different parts of the country, which eventually increases the burden of the already fragile health system in Bangladesh due to the increasing morbidity and fatality rates of COVID-19. Moreover, it can also affect the health system's epidemiological response to other infectious diseases, as it happened in other countries in the world, where the uncontrolled increase in the contagion curve has impaired coping with other diseases that also affected the country [10–12]. Monitoring is also required for assessing the effectiveness of vaccines against the circulating virus.

At this time, investing in establishing a systematic genetic surveillance for SARS-CoV-2 in different regions of the country is necessary to aid healthcare providers in updating clinical practice guidelines. Ultimately, a genetic surveillance program as a public health strategy should be swiftly implemented and realistic based on the capacity of the national infrastructure. Data need to be publicly available to drive real-time decision-making, enhance transparency, and fuel relevant research by national and international institutions. Therefore, collaboration between scientists and stakeholders is essential to ensure the rapid implementation of necessary measures.

## Data Availability

Not applicable.

## Abbreviations

COVID-19: Coronavirus disease 2019
SARS-CoV-2: Severe acute respiratory syndrome coronavirus 2
VOC: Variants of concern
CDC: Centers for Disease Control and Prevention
RT-PCR: Reverse transcription-polymerase chain reaction
